# Supplementary figures and images for: Neuroinflammation in neuronopathic Gaucher disease: Role of microglia and NK cells, biomarkers, and response to substrate reduction therapy
Source: eLife. 2022 Aug 16;11:e79830. doi: 10.7554/eLife.79830 (PMC9381039; doi:10.7554/eLife.79830)

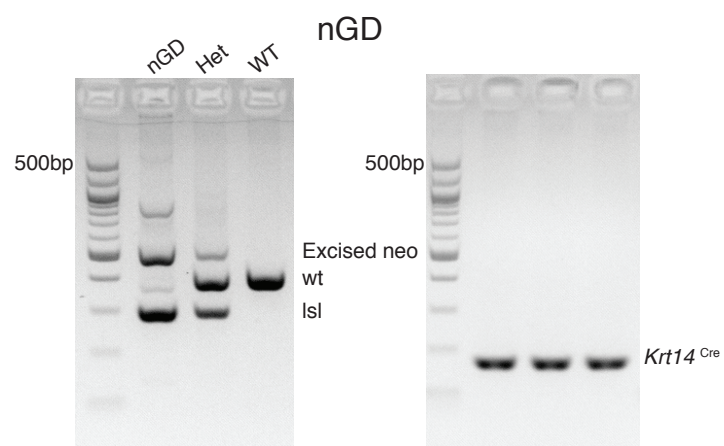

Supplement: Figure 2—figure supplement 1—source data 1. — The lsl (lox-stop-lox) cassette in nGD mice (left panel) was removed through to Krt14cre expression (right panel). [file elife-79830-fig2-figsupp1-data1.pdf]

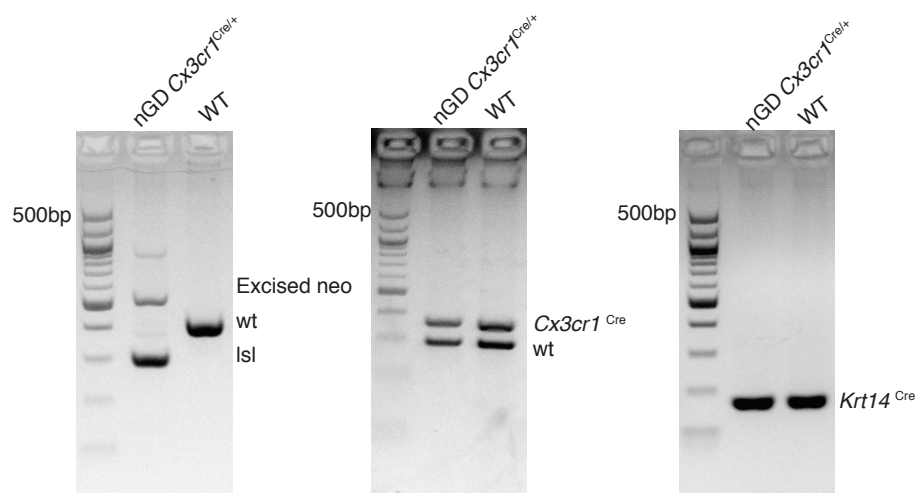

*nGD CX3Cr1<sup>Cre/+</sup>*

Supplement: Figure 2—figure supplement 1—source data 2. [file elife-79830-fig2-figsupp1-data2.pdf]

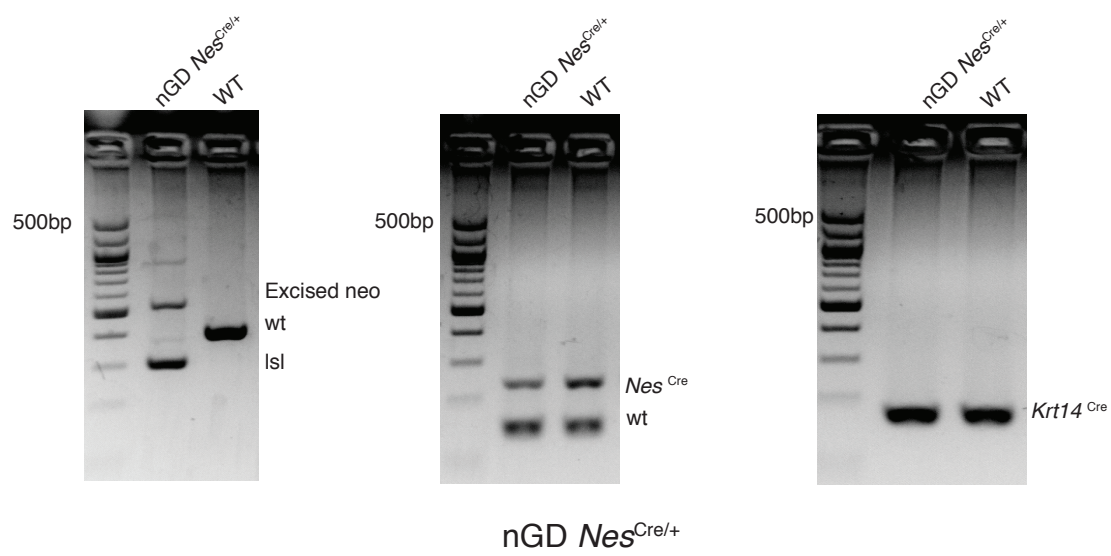

Supplement: Figure 2—figure supplement 1—source data 3. [file elife-79830-fig2-figsupp1-data3.pdf]

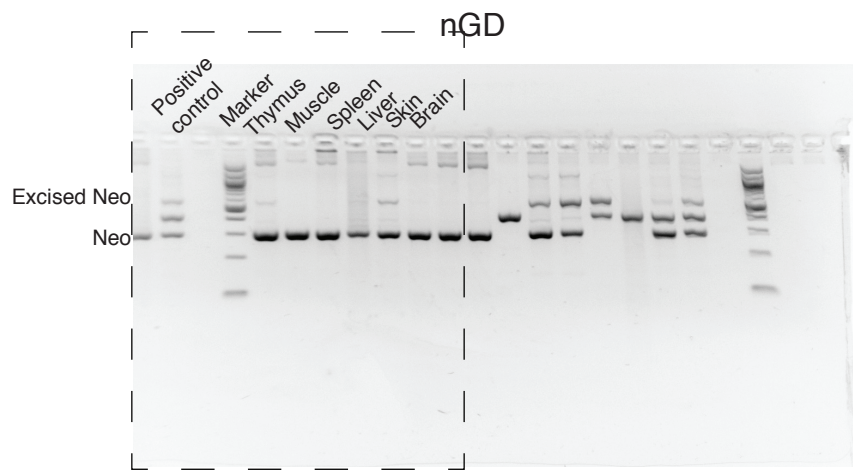

Supplement: Figure 2—figure supplement 1—source data 4. [file elife-79830-fig2-figsupp1-data4.pdf]

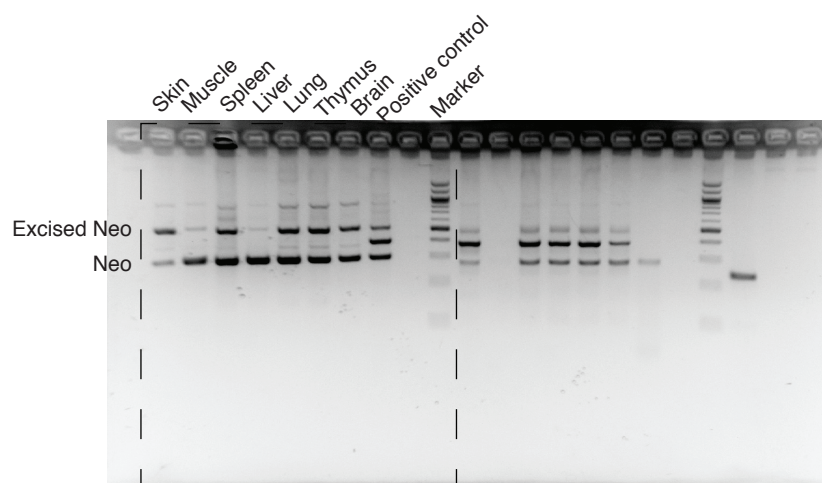

nGD *CX3Cr1*<sup>Cre/+</sup>

Supplement: Figure 2—figure supplement 1—source data 5. [file elife-79830-fig2-figsupp1-data5.pdf]

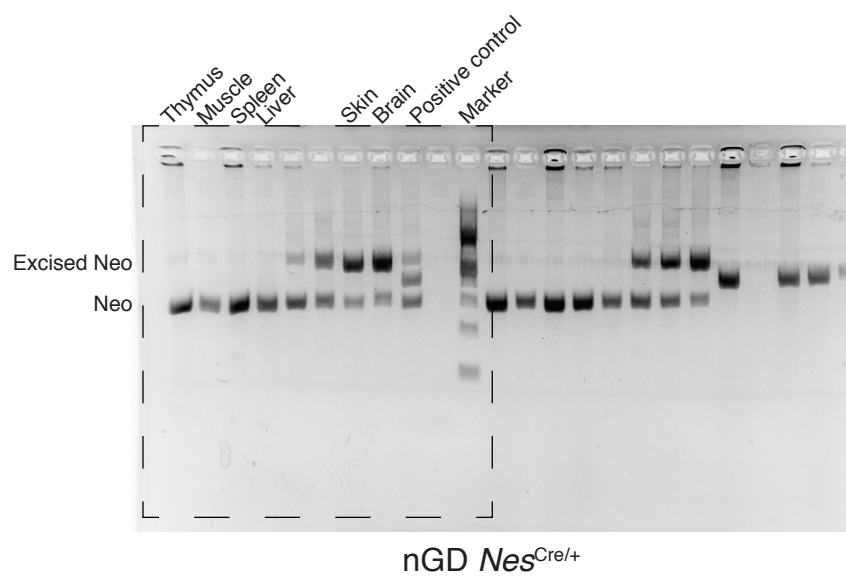

Supplement: Figure 2—figure supplement 1—source data 6. [file elife-79830-fig2-figsupp1-data6.pdf]

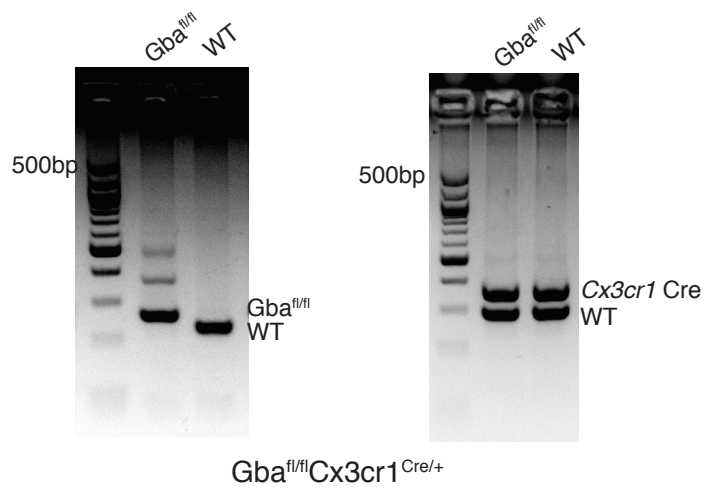

Supplement: Figure 3—figure supplement 1—source data 1. [file elife-79830-fig3-figsupp1-data1.pdf]
